# Exponents of the one-term Ogden model: insights from simple shear

**DOI:** 10.1098/rsta.2021.0328

**Published:** 2022-10-17

**Authors:** Cornelius O. Horgan, Jeremiah G. Murphy

**Affiliations:** ^1^ School of Engineering and Applied Science, University of Virginia, Charlottesville, VA 22904, USA; ^2^ Department of Mechanical Engineering, Dublin City University, Glasnevin, Dublin D09 W6Y4, Ireland; ^3^ School of Mathematics, Statistics and Applied Mathematics, National University of Ireland Galway, University Road, Galway, Ireland

**Keywords:** incompressibility, nonlinear elasticity, Ogden invariants, simple shear

## Abstract

Isotropic one-term Ogden models are widely used to predict the mechanical response of both incompressible elastomers and soft tissue. Even though the exponent might be chosen to yield excellent agreement with some aspects of mechanical response, there is no guarantee that these models will be physically realistic in all situations. We show here that, in particular, the predictions of models with either negative or large positive exponents do not seem physically realistic in simple shear. The mechanical response of materials in shear should be physically realistic to ensure rational and reliable predictions for complex geometries and boundary conditions. We suggest that for problematic values of exponents of one-term models that extra Ogden invariants should necessarily be included in the model.

This article is part of the theme issue ‘The Ogden model of rubber mechanics: Fifty years of impact on nonlinear elasticity’.

## 1. Introduction

The model of the mechanical response of incompressible elastomers introduced by Ogden [1] represents a paradigm shift away from models based essentially on polynomial functions of the strain invariants introduced by Rivlin [2]. Ogden proposed use of sums of what are now called the Ogden invariants, defined by $\Phi (n)=(\lambda_{1}^{n}+\lambda_{2}^{n}+\lambda_{3}^{n})/n$, where the principal stretches $\lambda_{1},\lambda_{2},\lambda_{3}$ are used as independent variables subject to the incompressibility constraint $\lambda_{1}\lambda_{2}\lambda_{3}=1$. This approach has proved enormously influential and the proof of its versatility can be found in the wide range of its applications. Although, in general, Ogden’s approach is typically implemented with a finite sum of invariants, for ease of exposition here only one-term Ogden models are considered. It should be noted, however, that even these simple models have proved themselves as effective mathematical models in a wide range of applications, as can be seen in table 1. This list is not intended to be exhaustive and there is an emphasis on more recent applications. Furthermore, in many of these studies, dynamic loading and viscoelastic effects were of main concern. Even this brief overview of the application of the one-term model reveals a division of application between soft tissue and elastomers, with the soft tissue typically being modelled with exponents that have a much higher absolute value. Another noteworthy feature is the large range of the proposed exponent for what is essentially the same type of material in many cases. This raises the issue of which values of the exponent are optimal in some sense.

**Table 1.** Applications of the one-term Ogden model.

material	exponent	reference
passive myocardium	18	Bogen & McMahon [3]
porcine and human skin	9, 12	Shergold *et al.* [4]
silicone rubbers	2.5, 3	Shergold *et al.* [4]
porcine adipose tissue	23	Comley & Fleck [5]
porcine brain tissue	2.766, 4, 6	Rashid *et al.* [6–8]
human brain tissue	−8.05	Mihai *et al.* [9]
human brain tissue	(−39.9,−15.2)	Budday *et al.* [10]
human brain tissue	(−35.83,−17.99)	Budday *et al.* [11]
cranial pia mater	(0.094, 1.702)	Li *et al.* [12]
thrombus mimic	(11.23, 16.38)	Sugerman *et al.* [13]
human adipose tissue	5.633, 13.361	Sun *et al.* [14]
porcine adipose tissue	6.670, 15.122	Sun *et al.* [14]

Although the widespread use of one-term Ogden models is obvious from table 1, caution must be exercised in the use of these models; although the exponent might have been chosen to yield excellent agreement with some aspects of mechanical response, there is no guarantee that these models will be robust in all situations. The effect of the exponent on the mechanical response of one-term Ogden models is of interest here, with the canonical deformation of simple shear chosen to illustrate what are considered here to be some deficiencies in one-term Ogden models with either negative or large positive exponents. The simplest conceptualization of shear is when platens are fixed to parallel faces of a cuboid specimen and the platens are moved parallel to each other. An instructive elegant illustration of this is given by Rashid *et al.* [6], who performed simple shear experiments on porcine brain tissue; the approximate homogeneous nature of the deformation is clear from their fig. 4, even for moderate amounts of shear on relatively soft materials such as brain tissue. The classical homogeneous deformation embodied in the semi-inverse approach to the modelling of simple shear of Rivlin [2] is therefore a reasonable approximation to the actual mechanical response. It should be noted, however, that other experiments have demonstrated a considerable degree of inhomogeneity. For example, Budday *et al.* [15] provided FE simulations of their experiments on brain tissue that show highly inhomogeneous response in shear. Furthermore, these authors found that large absolute values of the Ogden parameter n lead to unrealistic high shear stresses. Similar findings have been reported in Zhao *et al.* [16].

Although the kinematics of simple shear are well understood, modelling difficulties arise because there is a fundamental ambiguity in determining the pressure term in the normal stresses and different seemingly well-motivated assumptions yield qualitatively different normal stress distributions. Three such assumptions are considered here:

1. Rivlin [2] proposed that plane stress conditions could be assumed in order to determine the pressure and hence the stress distribution. This is, by far, the most commonly used approach to simple shear.
2. An alternative approach was first suggested by Rivlin [2] (although not subsequently used by him), namely that the normal component of the traction on the inclined faces should be identically zero. This has been advocated more emphatically as a physically realistic assumption by Gent *et al.* [17] and was examined in detail by Horgan & Murphy [18]. Both of these assumptions are well motivated physically as in simple shear experiments the specimens are in a state of plane stress, and there is no applied traction on the inclined faces. However, they are incompatible in general.
3. Yet another proposal to determine the pressure term can be found in Horgan & Murphy [18]. They required that the stress distribution for a perfectly incompressible material be the same as that for a slightly compressible counterpart which was shown to be equivalent to requiring that the three-dimensional hydrostatic Cauchy stress for simple shear be identically zero. Although not as immediately self-evident as the other two, this should also be physically realistic as all materials are to some extent compressible.

Each criterion is separately adopted here to determine, if possible, the existence of values of the Ogden exponent that are compatible with the other two assumptions. It is shown that the exponent n=6 is optimal in this sense. This suggests that using this exponent as a seed value for nonlinear optimization techniques or its inclusion as a term in a finite sum of Ogden invariants might yield reliable model predictions. With optimality comes its converse: those values of the exponents that are seemingly not compatible with the physically realistic modelling of simple shear using a one-term Ogden model. It would appear that negative and large positive exponents are not consistent with physically realistic behaviour in simple shear. Even though one-term Ogden models with these exponents are consistent with data from other experiments, the analysis presented here suggests that these models are perhaps too simple in form to capture the important normal stress effects in simple shear. Thus, as in the majority of applications of these models, additional terms are needed to form a fully realistic model.

## 2. Modelling simple shear using principal stretches

The preliminaries given here follow the treatment of Horgan & Murphy [18] and are summarized below. The deformation known as simple shear has the mathematical representation

2.1 $$x_{1}=X_{1}+\kappa X_{2},\;x_{2}=X_{2}\;\mathrm{and}\;x_{3}=X_{3},$$

where $\left(X_{1},X_{2},X_{3}\right)$ and $\left(x_{1},x_{2},x_{3}\right)$ denote the Cartesian coordinates of a typical particle before and after deformation respectively and κ>0 is an arbitrary dimensionless constant called the amount of shear. Within the context of nonlinear elasticity, this problem was first considered by Rivlin [2]. As shown in Horgan & Murphy [18], the in-plane principal stretches $\lambda_{1},\lambda_{2}$ are determined from the quartic

2.2 $$\lambda^{4}-\lambda^{2}(2+\kappa^{2})+1=0,$$

while the out-of-plane stretch is $\lambda_{3}=1$. As noted by [19], this quartic can be rewritten as

2.3 $$\lambda^{2}+\frac{1}{\lambda^{2}}-2-\kappa^{2}={\left(\lambda -\frac{1}{\lambda }\right)}^{2}-\kappa^{2}=0,$$

and thus yields the following quadratic for λ:

2.4 $$\lambda -\frac{1}{\lambda }=\pm \kappa .$$

Without loss of generality define the two in-plane stretches in terms of the amount of shear κ as follows:

2.5 $$\lambda_{1}=\frac{\kappa +\sqrt{4+\kappa^{2}}}{2}\;\mathrm{and}\;\lambda_{2}=\frac{1}{\lambda_{1}}=\frac{-\kappa +\sqrt{4+\kappa^{2}}}{2}.$$

It is shown in Horgan & Murphy [18] that the in-plane Cauchy stresses for simple shear are given by

2.6 $$\begin{array}{rl} T_{11} & \;=-p+\frac{\lambda_{1}^{3}}{1+\lambda_{1}^{2}}\frac{\partial W}{\partial \lambda_{1}}+\frac{\lambda_{2}^{3}}{1+\lambda_{2}^{2}}\frac{\partial W}{\partial \lambda_{2}},\\ T_{22} & \;=-p+\frac{\lambda_{1}}{1+\lambda_{1}^{2}}\frac{\partial W}{\partial \lambda_{1}}+\frac{\lambda_{2}}{1+\lambda_{2}^{2}}\frac{\partial W}{\partial \lambda_{2}}\\ \mathrm{and}\;T_{12} & \;=\frac{\lambda_{1}^{2}}{1+\lambda_{1}^{2}}\frac{\partial W}{\partial \lambda_{1}}-\frac{\lambda_{2}^{2}}{1+\lambda_{2}^{2}}\frac{\partial W}{\partial \lambda_{2}}. \end{array}\}$$

The out-of-plane axial stress is simply

2.7 $$T_{33}=-p+\frac{\partial W}{\partial \lambda_{3}}.$$

As noted by Rivlin [2], in general, surface tractions have to be applied to the inclined faces of the deformed specimen in order to maintain the deformation (2.1). On resolving these tractions into components tangential and normal to the surfaces on which they act in their deformed state (denoted by S and N, respectively), one finds that (e.g. Rivlin [2], Horgan & Murphy [18])

2.8 $$\begin{array}{rl} S & \;=\frac{\lambda_{1}^{4}}{1+\lambda_{1}^{6}}\frac{\partial W}{\partial \lambda_{1}}-\frac{\lambda_{2}^{4}}{1+\lambda_{2}^{6}}\frac{\partial W}{\partial \lambda_{2}}\\ & \;=\frac{\lambda_{1}^{2}(\lambda_{1}^{2}+1)}{\lambda_{1}^{6}+1}T_{12}\\ \mathrm{and}\;N & \;=-p+\frac{\lambda_{1}}{1+\lambda_{1}^{6}}\frac{\partial W}{\partial \lambda_{1}}+\frac{\lambda_{2}}{1+\lambda_{2}^{6}}\frac{\partial W}{\partial \lambda_{2}}\\ & \;=T_{22}-\frac{\lambda_{1}(\lambda_{1}^{4}-1)}{\lambda_{1}^{6}+1}T_{12}. \end{array}\}$$

We restrict attention in this paper to the one-term Ogden model

2.9 $$W=\frac{2\mu }{n^{2}}(\lambda_{1}^{n}+\lambda_{2}^{n}+\lambda_{3}^{n}-3),\;n\neq 0,$$

where μ is the infinitesimal shear modulus. The shear stress and traction are given by

2.10 $$T_{12}=\frac{2\mu }{n}\left(\frac{\lambda^{n+1}-\lambda^{1-n}}{1+\lambda^{2}}\right)\;\mathrm{and}\;S=\frac{2\mu }{n}\left(\frac{\lambda^{n+3}-\lambda^{3-n}}{1+\lambda^{6}}\right),$$

where for convenience we set $\lambda_{1}\equiv \lambda ,\lambda_{2}\equiv \lambda^{-1}$. For the special case of n=2 so that (2.9) reduces to the neo-Hookean model, these results are examined in detail in Horgan & Murphy [18]. Another special case of (2.9) is that when n=1, in which case one obtains the model due to Varga [20]. The shear stress will be assumed to have been matched with experimental data. It is an odd function of the exponent n. The mechanical response in simple shear is shear-stiffening if $T_{12}^{\prime }(\lambda )>0$, with a transition to shear-softening at those values of stretch for which $T_{12}^{\prime }(\lambda )=0$ or equivalently

2.11 $$(n+1)(\lambda^{n}+\lambda^{2-n})+(n-1)(\lambda^{n+2}+\lambda^{-n})=0.$$

If |n|≥1, there are clearly no solutions for λ>0; if |n|<1 there are two solutions, one compressive and one tensile.

## 3. Plane stress formulation for the one-term Ogden model

The analysis of simple shear illustrates one of the problems that can arise when using the classical semi-inverse approach to solving boundary value problems in nonlinear elasticity: there are ambiguities arising from the determination of the arbitrary hydrostatic pressure term in the normal stresses for the case of an incompressible isotropic hyperelastic material. If one were to try to reproduce simple shearing in a laboratory, one would not expect to have to apply forces in the out-of-plane direction nor on the sloping faces of the specimen in shear. There is therefore a dilemma: Which boundary condition to enforce? The approach adopted here is to enforce one boundary condition and then seek to minimize the effect of this on the other through a judicious choice of the exponent of the Ogden invariant.

First therefore we impose the plane stress condition that $T_{33}=0$. This is the most commonly used assumption when discussing simple shear and, implicitly, was the approach favoured by Rivlin [2]. With the arbitrary pressure term now determined from (2.7) to be $\partial W/\partial \lambda_{3}$, the in-plane normal stresses are therefore determined from (2.6) to be

3.1 $$\begin{array}{rl} T_{11} & \;=-\frac{\partial W}{\partial \lambda_{3}}+\frac{\lambda_{1}^{3}}{1+\lambda_{1}^{2}}\frac{\partial W}{\partial \lambda_{1}}+\frac{\lambda_{2}^{3}}{1+\lambda_{2}^{2}}\frac{\partial W}{\partial \lambda_{2}}\\ \mathrm{and}\;T_{22} & \;=-\frac{\partial W}{\partial \lambda_{3}}+\frac{\lambda_{1}}{1+\lambda_{1}^{2}}\frac{\partial W}{\partial \lambda_{1}}+\frac{\lambda_{2}}{1+\lambda_{2}^{2}}\frac{\partial W}{\partial \lambda_{2}}, \end{array}\}$$

with the normal traction on the inclined faces given by

$$N=-\frac{\partial W}{\partial \lambda_{3}}+\frac{\lambda_{1}}{1+\lambda_{1}^{6}}\frac{\partial W}{\partial \lambda_{1}}+\frac{\lambda_{2}}{1+\lambda_{2}^{6}}\frac{\partial W}{\partial \lambda_{2}}.$$

For the one-term Ogden model (2.9), the normal stresses are

3.2 $$T_{11}=\frac{2\mu }{n}\left(\frac{\left(\lambda^{n}-1)(\lambda^{2}-\lambda^{-n}\right)}{1+\lambda^{2}}\right)\;\mathrm{and}\;T_{22}=\frac{2\mu }{n}\left(\frac{\left(\lambda^{n}-1)(1-\lambda^{2-n}\right)}{1+\lambda^{2}}\right),$$

with the normal traction on the inclined faces given by

3.3 $$N=\frac{2\mu }{n}\left(\frac{\left(\lambda^{n}-1)(1-\lambda^{6-n}\right)}{1+\lambda^{6}}\right).$$

### (a) Traction on the sloping faces

Normal tractions are rarely, if ever, applied to the sloping faces in simple shear experiments. In order to assess the effect of not applying a normal traction, the relative size of N with respect to the shearing stress will be considered where

3.4 $$\hat{N}\equiv \frac{N}{T_{12}}=\frac{\lambda^{n}-\lambda^{6}}{\lambda (1+\lambda^{4}-\lambda^{2})(\lambda^{n}+1)}.$$

Ideally, then in order to match experiments, one would want

3.5 $$|\hat{N}|\ll 1.$$

**Figure 1.**
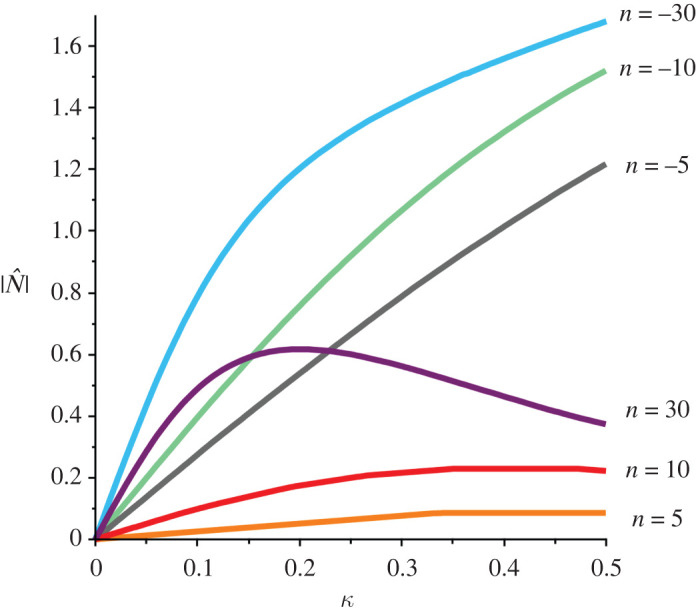
Plots of the relative normal traction (3.4) versus amount of shear κ for the Ogden exponents n. Large normal tractions are predicted to be necessary to maintain homogeneity for negative and for large positive exponents. (Online version in colour.)

Trivially $\hat{N}=0\;⟺\;n=6$, which therefore yields an optimal value for the exponent of the Ogden invariant. This optimality is very sensitive with significant deviations from (3.5), occurring for even small amounts of shear when n≠6. This is illustrated in figure 1 where it is shown that relatively significant amounts of normal traction need to be supplied to maintain homogeneity of the specimen when the exponent is negative or when the exponent is large and positive (n>10) even for the moderate amounts of shear considered here. It follows then that one-term models with negative or large positive exponents are not compatible with specimens in simple shear being deformed homogeneously in the absence of normal tractions on the sloping face. It would seem that additional Ogden terms need to be incorporated into the modelling process in order to capture physically realistic behaviour.

### (b) The hydrostatic stress

The hydrostatic stress H for plane stress conditions is given by

**Figure 2.**
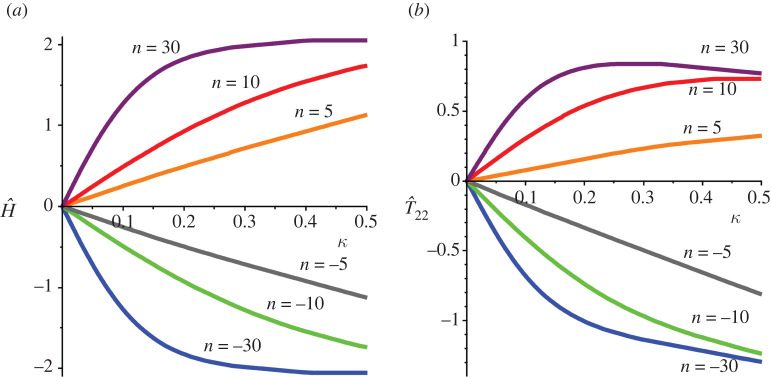
(*a*) Plots of the relative *hydrostatic* stress versus amount of shear for different Ogden exponents. Large exponents result in large hydrostatic stresses for relatively small amounts of shear. (*b*) Plots of the relative normal stress versus amount of shear for different Ogden exponents in order to assess the Poynting effect. Note the seemingly unrealistic response for large negative exponents. (Online version in colour.)

3.6 $$H=T_{11}+T_{22}=\frac{2\mu }{n}(\lambda^{n}+\lambda^{-n}-2),$$

so that the hydrostatic stress is tensile for n>0 and compressive for n<0. Plots of the relative hydrostatic stress $\hat{H}(n)\equiv H/|T_{12}|$ versus the amount of shear κ are given in figure 2*a*, where it is obvious that large exponents (≥5 here) result in large hydrostatic stresses for relatively small amounts of shear. This is problematic for these models as there is potentially a large discrepancy between the theoretical predictions based on perfect incompressibility and the predictions of finite-element simulations usually carried out for slightly compressible versions for ostensibly the same material in simple shear, noting that for agreement between the two approaches the hydrostatic stress should be small. This will be explored in detail elsewhere. The hydrostatic stress has also an important physical interpretation: the classical experiments of Gent & Lindley [21] have identified large tensile hydrostatic stress as a significant contributor to the damage and failure of rubbers. The failure of isotropic rubber blocks owing to large hydrostatic stress in simple shear was discussed in Gent *et al.* [17], where it was suggested that a possible mechanism of failure was cavitation. Thus one-term Ogden models with large positive exponents could be be useful in modelling cavitation in simple shear. However, to the best of our knowledge, cavitation in shear has not been observed experimentally.

### (c) The Poynting effect

Finally, the well-known Poynting effect is considered. The stress normal to the direction of shearing $T_{22}$ is given by (3.2)${\;}_{2}$, which is negative for n<2 so that the classical Poynting effect is predicted. For n>2, the reverse Poynting effect occurs, i.e. the specimen will contract in the normal direction in shear $\left(T_{22}>0\right)$. For the special case n=2, $T_{22}=0$ so that we recover the well-known result that there is no Poynting effect in simple shear for neo-Hookean materials. The relative normal stress ${\hat{T}}_{22}\equiv (T_{22}/|T_{12}|)$ is plotted in figure 2*b* for sample exponents. Negative exponents predict a large positive Poynting effect for the moderate amounts of shear considered here.

The normal stress is rarely measured in simple shear. There are two notable exceptions that allow us to judge which range of exponents are likely to yield physically realistic results. Janmey *et al.* [22] showed that networks of semiflexible biopolymers exhibit a reverse Poynting effect when sheared between two plates and crucially for the purposes here showed that these negative normal stresses *can be as large as the shear stress*. It should be noted that the experiments reported by Janmey *et al.* [22] were performed using a torsional rheometer and the materials considered were likely anisotropic. Nevertheless, an extrapolation of these results suggests the restriction that

**Figure 3.**
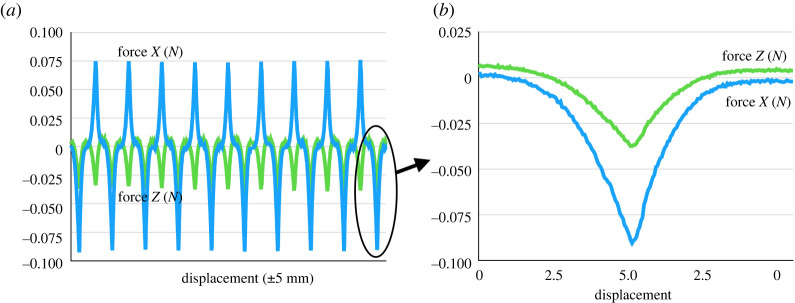
Plots of the full shear (force X) and normal force (force Z) data in Newtons of Sugerman *et al.* [13] versus displacement for sample 1 of a thrombus mimic with a calcium chloride concentration of 40 mM coagulated for 60 min are given in (*a*). The fully preconditioned data used in the analysis here are highlighted. These preconditioned data are enlarged in (*b*). (Online version in colour.)

3.7 $$|{\hat{T}}_{22}|<1,$$

corresponds to realistic behaviour, which would suggest that one-term models with large negative exponents are not viable and should be supplemented with extra terms. Further evidence for this can be found in Sugerman *et al.* [13], who developed an *in vitro* thrombus mimic and tested this mimic under large deformation simple shear. Two notable features of their work are that thrombus mimics exhibit a negative Poynting effect and of the three hyperelastic constitutive models that were tested, the one-term Ogden model provided the best fits to both shear stress and normal stress. They conducted simple shear experiments on cubic specimens of side 10 mm by symmetrically displacing the top plate of a custom simple shear testing device by ±5 mm in each direction at a rate of $0.1\;\text{mm}\;{\text{s}}^{-1}$ for 10 consecutive cycles. During the experiments, the distance between top and bottom plates was held constant. Simultaneously, force was measured in three directions using a 2 N capacity triaxial load cell (0.5% accuracy). A complete set of results can be found at https://dataverse.tdl.org/dataverse/STBML. The 40_60_1 dataset will be used here for illustrative purposes. This dataset is represented graphically in figure 3*a*, where the fully preconditioned data are highlighted. These preconditioned data are shown in more detail in figure 3*b*. It is easily shown that these data are consistent with the empirical restriction (3.7).

### (d) Summary for plane stress

For the moderate range of shearing strains considered here (±50%), it has been shown that negative exponents for the one-term Ogden model predict that large normal tractions on the sloping faces are required to maintain homogeneity of these materials under simple shear; simple shear experiments are usually performed without these tractions. Large compressive hydrostatic stress is predicted for these exponents; such stress means that predictions of finite-element simulations based on slight compressibility models are likely to be significantly different from those predicted here. Finally, negative exponents predict a large classical Poynting effect.

For large positive exponents (taken here to be exponents greater than 10), large normal tractions on the inclined faces are again required to maintain homogeneity of samples in simple shear. Large tensile hydrostatic stress is predicted for the moderate amounts of shear with cavitation damage likely. As far as we are aware, such damage has not been observed experimentally. Again there are likely to be major differences in predictions between the results here and those obtained in finite element simulations. Finally, a large reverse Poynting effect is predicted for these exponents, with samples predicted to contract significantly in simple shear unless constrained otherwise.

The value n=6 is optimal in simple shear in the sense that normal tractions on the inclined faces are identically zero, with a moderate tensile hydrostatic stress and moderate positive normal stress predicted for moderate amounts of shear.

## 4. Zero normal traction on the inclined faces

One alternative to imposing plane stress conditions in simple shear was first suggested by Rivlin [2] (although not subsequently used by him), namely that the normal component of the traction on the inclined faces should be identically zero. This has been advocated more emphatically as a physically realistic assumption by Gent *et al.* [17] and has been further investigated in Horgan & Murphy [18]. If the zero normal traction condition is enforced, then setting N≡0 in (2.8)${\;}_{2}$ determines the pressure to be

$$p=\frac{\lambda_{1}}{1+\lambda_{1}^{6}}\frac{\partial W}{\partial \lambda_{1}}+\frac{\lambda_{2}}{1+\lambda_{2}^{6}}\frac{\partial W}{\partial \lambda_{2}}.$$

The normal stress components are therefore

4.1 $$\begin{array}{rl} T_{11} & \;=\frac{\lambda_{1}(\lambda_{1}^{2}-1)(\lambda_{1}^{4}+1)}{1+\lambda_{1}^{6}}\frac{\partial W}{\partial \lambda_{1}}+\frac{\lambda_{2}(\lambda_{2}^{2}-1)(\lambda_{2}^{4}+1)}{1+\lambda_{2}^{6}}\frac{\partial W}{\partial \lambda_{2}}\\ T_{22} & \;=\frac{\lambda_{1}^{3}(\lambda_{1}^{2}-1)}{1+\lambda_{1}^{6}}\frac{\partial W}{\partial \lambda_{1}}+\frac{\lambda_{2}^{3}(\lambda_{2}^{2}-1))}{1+\lambda_{2}^{6}}\frac{\partial W}{\partial \lambda_{2}}\\ \mathrm{and}\;T_{33} & \;=-\frac{\lambda_{1}}{1+\lambda_{1}^{6}}\frac{\partial W}{\partial \lambda_{1}}-\frac{\lambda_{2}}{1+\lambda_{2}^{6}}\frac{\partial W}{\partial \lambda_{2}}+\frac{\partial W}{\partial \lambda_{3}}. \end{array}\}$$

If again for convenience we set $\lambda_{1}\equiv \lambda$, with $\lambda_{2}=1/\lambda$, then

4.2 $$\begin{array}{rl} T_{11} & \;=\frac{\lambda (\lambda^{2}-1)(\lambda^{4}+1)}{1+\lambda^{6}}\left(\frac{\partial W}{\partial \lambda_{1}}-\frac{1}{\lambda^{2}}\frac{\partial W}{\partial \lambda_{2}}\right)=\frac{\lambda (\lambda^{2}-1)(\lambda^{4}+1)}{1+\lambda^{6}}w^{\prime }(\lambda ),\\ T_{22} & \;=\frac{\lambda^{3}(\lambda^{2}-1)}{1+\lambda^{6}}\left(\frac{\partial W}{\partial \lambda_{1}}-\frac{1}{\lambda^{2}}\frac{\partial W}{\partial \lambda_{2}}\right)=\frac{\lambda^{3}(\lambda^{2}-1)}{1+\lambda^{6}}w^{\prime }(\lambda )\\ \mathrm{and}\;T_{33} & \;=-\frac{\lambda }{1+\lambda^{6}}\frac{\partial W}{\partial \lambda_{1}}-\frac{\lambda^{5}}{1+\lambda^{6}}\frac{\partial W}{\partial \lambda_{2}}+\frac{\partial W}{\partial \lambda_{3}}, \end{array}\}$$

where

4.3 $$w(\lambda )=W\left(\lambda ,\frac{1}{\lambda },1\right).$$

The hydrostatic stress is therefore

$$H=T_{11}+T_{22}+T_{33}=\frac{\lambda (\lambda^{6}-2)}{1+\lambda^{6}}\frac{\partial W}{\partial \lambda_{1}}+\frac{1-2\lambda^{6}}{\lambda (1+\lambda^{6})}\frac{\partial W}{\partial \lambda_{2}}+\frac{\partial W}{\partial \lambda_{3}}.$$

The shear stresses and traction can be written as

4.4 $$T_{12}=\frac{\lambda^{2}}{1+\lambda^{2}}w^{\prime }(\lambda )\;\mathrm{and}\;S=\frac{\lambda^{4}}{1+\lambda^{6}}w^{\prime }(\lambda ).$$

**Figure 4.**
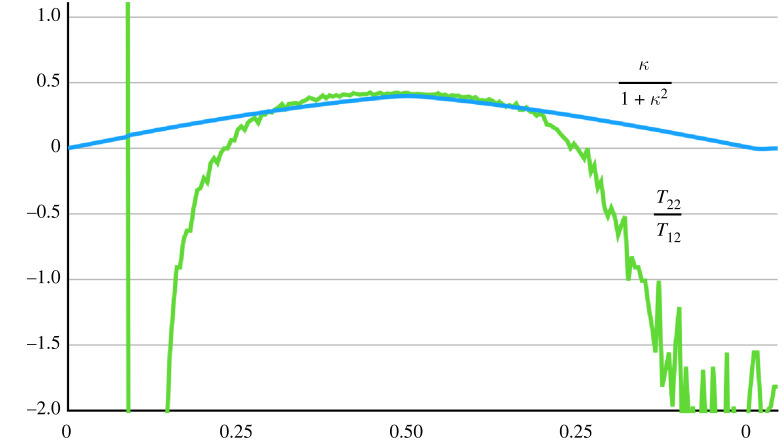
Verification of (4.5)${\;}_{2}$ for moderate amounts of oscillating shear (experimental data due to Sugerman *et al.* [13]). The large discrepancies occur close to the undeformed configuration for which the applied forces are small leading to the errors in the calculation of the stress ratio $T_{22}/T_{12}$. (Online version in colour.)

Therefore, if N≡0, then the following quasi-universal relations hold:

4.5 $$\begin{array}{rl} \frac{T_{11}}{T_{12}} & \;=\frac{\left(\lambda^{2}-1)(\lambda^{4}+1\right)}{\lambda (1+\lambda^{4}-\lambda )}=\frac{\kappa (2+\kappa^{2})}{1+\kappa^{2}}\\ \mathrm{and}\;\hat{T}\equiv \frac{T_{22}}{T_{12}} & \;=\frac{\lambda (\lambda^{2}-1)}{1+\lambda^{4}-\lambda }=\frac{\kappa }{1+\kappa^{2}}, \end{array}\}$$

thus recovering the results of Horgan & Murphy [18]. These relations are called quasi-universal since they hold only for the special choice for p which does depend on W. The second of these has a practical significance as both $T_{12},\;T_{22}$ can be measured experimentally. The very limited experimental evidence available supports this relation for moderate amounts of shear. Considering again the 40_60_1 dataset of Sugerman *et al.* [13] introduced in the last section, both $T_{22}/T_{12}$ and $\kappa /\left(1+\kappa^{2}\right)$ are plotted in figure 4 versus the oscillating amount of shear. There is a close correspondence between the two curves for moderate amounts of shear, with the large differences occurring close to the undeformed configuration explained by the difficulty in accurately measuring the stress ratio for small shear using the available load cells.

There is a further use of (4.5)${\;}_{2}$ in assessing the validity of constitutive assumptions: since $\max(\kappa /1+\kappa^{2})=1/2$ at κ=1, it seems reasonable to require that *all* models satisfy

4.6 $$|\hat{T}|<0.5,$$

which is more restrictive on the allowable range of the stress ratio than the empirical restriction (3.7) which was inferred from the experimental data of Janmey *et al.* [22]. The fact that many of the flexible gels tested by Janmey *et al.* [22] violate (4.6) suggest that many of these gels are not isotropic.

The out-of-plane stress $T_{33}$ and the hydrostatic stress H for the one-term Ogden model are, respectively

4.7 $$T_{33}=\frac{2\mu }{n}\left(\frac{\left(\lambda^{n}-1)(\lambda^{6-n}-1\right)}{1+\lambda^{6}}\right)$$

and

4.8 $$H=\frac{2\mu }{n}\left(1+\frac{\lambda^{n+6}-2\lambda^{n}+\lambda^{-n}-2\lambda^{6-n}}{1+\lambda^{6}}\right).$$

Note that the out-of-plane normal stress is the negative of the normal traction under plane stress conditions given in (3.3). Therefore, n=6 is again optimal in that for this exponent the out-of-plane stress is identically zero and the plots of $|\hat{N}|$ against amount of shear in figure 1 will be exactly the same as plots of $|T_{33}/T_{12}|$ here. Thus the observations following figure 1 are valid here as well with negative exponents leading to the requirement of large out-of-plane stresses needed to maintain simple shear, stresses which are never applied in practice.

**Figure 5.**
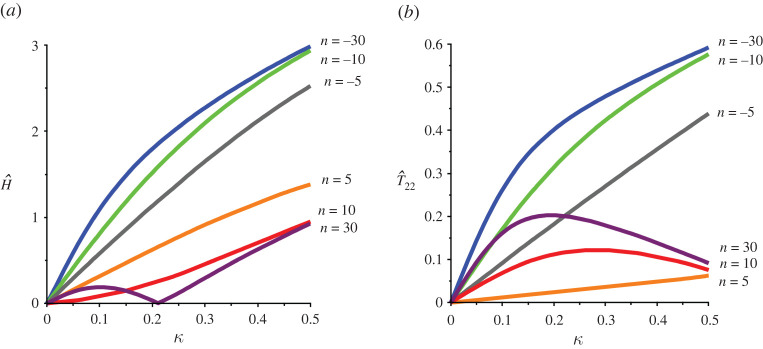
(*a*) Plots of the normalized hydrostatic stress $\hat{H}\equiv (H/|T_{12}|)$ versus amount of shear for different Ogden exponents for the case of zero normal traction on the inclined faces. Note the large tensile stresses for negative values of the exponent for moderate amounts of shear. (*b*) Plots of the normalized normal stress versus amount of shear for different Ogden exponents when the hydrostatic stress is assumed to be zero. (Online version in colour.)

It follows from (4.8) that the hydrostatic stress is tensile for all values of n. Plots of the normalized hydrostatic stress $\hat{H}\equiv (H/|T_{12}|)$ are given in figure 5*a*, where it can be clearly seen that negative exponents result in large tensile hydrostatic stress for even moderate amounts of shear.

### (a) The Poynting effect

The Poynting effect is now considered. It follows from (4.2) that for the one-term Ogden model

4.9 $$T_{22}=\frac{2\mu }{n}\left(\frac{\lambda^{2}(\lambda^{2}-1)\lambda^{-n}(\lambda^{2n}-1)}{1+\lambda^{6}}\right).$$

Therefore, $T_{22}>0$, the reverse Poynting effect in which specimens contract in simple shear, is predicted for all amounts of shear and for all exponents. For the special case of n=2, i.e. the neo-Hookean model, this result was obtained by Horgan & Murphy [18]. Recall that in the plane stress formulation described in §3, there was no Poynting effect in this case. The importance of determining the type of Poynting effect for constitutive modelling is illustrated by Destrade *et al.* [23], who verified experimentally that brain matter exhibits a classical Poynting effect, which is inconsistent with assuming a one-term Ogden model and no traction on the inclined faces. Thus more than one Ogden invariant would be needed to model the mechanical response of brain tissue, especially in light of the apparent homogeneity in sheared samples shown in fig. 4 of Rashid *et al.* [6].

## 5. Zero hydrostatic stress

The third alternative outlined in the Introduction will now be briefly examined. See Horgan & Murphy [18] for details of this approach. Assume now that the hydrostatic stress is zero. This ensures that the stress distribution in the isochoric deformation of simple shear of the incompressible material $W(\lambda_{1},\lambda_{2},\lambda_{3})$ and the slightly compressible material

$$W=f(J)+W(J^{-(1/3)}\lambda_{1},J^{-(1/3)}\lambda_{2},J^{-(1/3)}\lambda_{3}),\;J\equiv \lambda_{1}\lambda_{2}\lambda_{3},$$

favoured by commercial finite-element codes, are identical (Horgan & Murphy [18]). It follows from (3.1), (2.7) that the hydrostatic stress is zero if and only if

$$p=\frac{\lambda_{1}}{3}\frac{\partial W}{\partial \lambda_{1}}+\frac{\lambda_{2}}{3}\frac{\partial W}{\partial \lambda_{2}}+\frac{1}{3}\frac{\partial W}{\partial \lambda_{3}},$$

which determines the normal stresses and normal traction on the inclined faces to be

5.1 $$\begin{array}{rl} T_{11} & \;=\frac{\lambda_{1}(2\lambda_{1}^{2}-1)}{3(1+\lambda_{1}^{2})}\frac{\partial W}{\partial \lambda_{1}}+\frac{\lambda_{2}(2\lambda_{2}^{2}-1)}{3(1+\lambda_{2}^{2})}\frac{\partial W}{\partial \lambda_{2}}-\frac{1}{3}\frac{\partial W}{\partial \lambda_{3}},\\ T_{22} & \;=\frac{\lambda_{1}(2-\lambda_{1}^{2})}{3(1+\lambda_{1}^{2})}\frac{\partial W}{\partial \lambda_{1}}+\frac{\lambda_{2}(2-\lambda_{2}^{2})}{3(1+\lambda_{2}^{2})}\frac{\partial W}{\partial \lambda_{2}}-\frac{1}{3}\frac{\partial W}{\partial \lambda_{3}},\\ T_{33} & \;=-\frac{\lambda_{1}}{3}\frac{\partial W}{\partial \lambda_{1}}-\frac{\lambda_{2}}{3}\frac{\partial W}{\partial \lambda_{2}}+\frac{2}{3}\frac{\partial W}{\partial \lambda_{3}}\\ \mathrm{and}\;N & \;=\frac{\lambda_{1}(2-\lambda_{1}^{6})}{3(1+\lambda_{1}^{6})}\frac{\partial W}{\partial \lambda_{1}}+\frac{\lambda_{2}(2-\lambda_{2}^{6})}{3(1+\lambda_{2}^{6})}\frac{\partial W}{\partial \lambda_{2}}-\frac{1}{3}\frac{\partial W}{\partial \lambda_{3}}. \end{array}\}$$

For the one-term Ogden model of interest here these become

5.2 $$\begin{array}{rl} T_{11} & \;=\frac{2\mu }{3n}\left(\frac{2\lambda^{n+2}+2\lambda^{-n}-\lambda^{n}-\lambda^{2-n}-1-\lambda^{2}}{1+\lambda^{2}}\right),\\ T_{22} & \;=\frac{2\mu }{3n}\left(\frac{2\lambda^{2-n}+2\lambda^{n}-\lambda^{-n}-\lambda^{n+2}-1-\lambda^{2}}{1+\lambda^{2}}\right),\\ T_{33} & \;=\frac{2\mu }{3n}(2-\lambda^{n}-\lambda^{-n})\\ \mathrm{and}\;N & \;=\frac{2\mu }{3n}\left(\frac{2\lambda^{6-n}+2\lambda^{n}-\lambda^{-n}-\lambda^{n+6}-1-\lambda^{6}}{1+\lambda^{6}}\right). \end{array}\}$$

The shear stresses are still given by (2.10).

First note that ${\hat{T}}_{33}\equiv T_{33}/T_{12}=-\hat{H}/3$, where $\hat{H}$ is given in the second line following (3.6) and therefore the qualitative features of the out-of-plane stress can be assessed from consideration of figure 2. The conclusion is that the relative out-of-plane stress is within the range (−2/3,2/3). Similarly $\hat{N}\equiv N/T_{12}$ is equal to negative one-third of the hydrostatic stress of §4 and its qualitative features can be assessed in figure 5*a*, from which it can be concluded that large compressive normal tractions are required for negative exponents for the range of shear considered here. For the special case of a neo-Hookean material (n=2), the foregoing results are analysed in detail in Horgan & Murphy [18].

Finally, the Poynting effect is considered. It follows from (5.2)${\;}_{2}$ that $T_{22}<0$ for all exponents during simple shear and therefore only the classical Poynting effect where specimens expand in shear unless constrained is predicted. The relative in-plane normal stress ${\hat{T}}_{22}\equiv |T_{22}/T_{12}|$ is plotted in figure 5*b*, where it is seen that the restriction (4.6) proposed in §4 is satisfied for n > –5.

## 6. Concluding remarks

The seminal paper of Ogden [1] introduced a model for the mechanical response of incompressible elastomers where the principal stretches were used as independent variables instead of the strain invariants proposed by Rivlin [2]. Strain-energy densities using the basic Ogden invariants (powers of the principal stretches) are usually proposed as a finite sum of such invariants. The widespread implementation of this approach to model the mechanical response of rubber-like and biological materials is testament to its lasting influence. In recent years, a variety of applications to the response of elastomers and biotissues have been presented in the literature using just a one-term Ogden model with a single invariant and this has been our focus here.

We have examined the viability of such an approach in the special context of simple shear. The choice of the exponent in the one-term model was a particular focus. Three alternatives to the modelling of simple shear were investigated following the general treatment of Horgan & Murphy [18]. For each of these alternatives, the choice of the arbitrary hydrostatic pressure arising from the incompressibility constraint differ and so do the resulting stress distributions. The first alternative, and the most commonly adopted in the literature, was to assume a plane stress state and it was shown that the special value of the Ogden exponent n=6 satisfied the desirable condition that the slanted faces of the deformed specimen should be subjected to zero normal traction, as is usual in experimental configurations.

The second alternative was to assume zero normal traction of the inclined faces at the outset. It was then shown that, in general, an out-of-plane normal stress was required to maintain simple shear except again for the special value of the Ogden exponent n=6. The third alternative was to assume that the hydrostatic stress was zero, motivated by the expectation that in the bulk of the sample, the stress distributions for incompressible materials should be close to those for their slightly compressible counterparts. In this case, a non-zero out-of-plane stress and normal traction on the inclined faces were necessary to maintain simple shear. By contrast, it was shown that negative exponents and large positive exponents yield predictions of the mechanical response in simple shear that are not physically realistic.

As suggested by Gent *et al.* [17] and Horgan & Murphy [18] and examined in detail in Horgan & Murphy [24], these ambiguities in the formulation of simple shear for incompressible materials might be resolved by using a boundary-layer approach with the first two alternatives used near the lateral and inclined faces respectively and the third alternative employed in the bulk of the specimen.

## Data Availability

This article has no additional data.

## References

[1] Ogden RW. 1972 Large deformation isotropic elasticity–on the correlation of theory and experiment for incompressible rubberlike solids. Proc. R. Soc. Lond. A **326**, 565-584. (10.1098/rspa.1972.0026)

[2] Rivlin RS. 1948 Large elastic deformations of isotropic materials IV. Further developments of the general theory. Phil. Trans. R. Soc. Lond. A **241**, 379-397. (10.1098/rsta.1948.0024)

[3] Bogen DK, McMahon TA. 1979 Do cardiac aneurysms blow out? Biophys. J. **27**, 301-316. (10.1016/S0006-3495(79)85219-4) 262437 PMC1328586

[4] Shergold OA, Fleck NA, Radford D. 2006 The uniaxial stress versus strain response of pig skin and silicone rubber at low and high strain rates. Int. J. Impact Eng. **32**, 1384-1402. (10.1016/j.ijimpeng.2004.11.010)

[5] Comley K, Fleck N. 2012 The compressive response of porcine adipose tissue from low to high strain rate. Int. J. Impact Eng. **46**, 1-10. (10.1016/j.ijimpeng.2011.12.009)

[6] Rashid B, Destrade M, Gilchrist MD. 2013 Mechanical characterization of brain tissue in simple shear at dynamic strain rates. J. Mech. Behav. Biomed. Mater. **28**, 71-85. (10.1016/j.jmbbm.2013.07.017) 23973615

[7] Rashid B, Destrade M, Gilchrist MD. 2013 Influence of preservation temperature on the measured mechanical properties of brain tissue. J. Biomech. **46**, 1276-1281. (10.1016/j.jbiomech.2013.02.014) 23523381

[8] Rashid B, Destrade M, Gilchrist MD. 2014 Mechanical characterization of brain tissue in tension at dynamic strain rates. J. Mech. Behav. Biomed. Mater. **33**, 43-54. (10.1016/j.jmbbm.2012.07.015) 23127641

[9] Mihai LA, Budday S, Holzapfel GA, Kuhl E, Goriely A. 2017 A family of hyperelastic models for human brain tissue. J. Mech. Phys. Solids **106**, 60-79. (10.1016/j.jmps.2017.05.015)

[10] Budday S *et al.* 2017 Mechanical characterization of human brain tissue. Acta Biomater. **48**, 319-340. (10.1016/j.actbio.2016.10.036) 27989920

[11] Budday S, Sommer G, Haybaeck J, Steinmann P, Holzapfel GA, Kuhl E. 2017 Rheological characterization of human brain tissue. Acta Biomater. **60**, 315-329. (10.1016/j.actbio.2017.06.024) 28658600

[12] Li Y, Zhang W, Lu YC, Wu CW. 2020 Hyper-viscoelastic mechanical behavior of cranial pia mater in tension. Clin. Biomech. **80**, 105108. (10.1016/j.clinbiomech.2020.105108) 32736277

[13] Sugerman GP, Kakaletsis S, Thakkar P, Chokshi A, Parekh SH, Rausch MK. 2021 A whole blood thrombus mimic: constitutive behavior under simple shear. J. Mech. Behav. Biomed. Mater. **115**, 104216. (10.1016/j.jmbbm.2020.104216) 33486384

[14] Sun Z, Lee SH, Gepner BD, Rigby J, Hallman JJ, Kerrigan JR. 2021 Comparison of porcine and human adipose tissue loading responses under dynamic compression and shear: a pilot study. J. Mech. Behav. Biomed. Mater. **113**, 104112. (10.1016/j.jmbbm.2020.104112) 33010697

[15] Budday S, Ovaert TC, Holzapfel GA, Steinmann P, Kuhl E. 2020 Fifty shades of brain: a review on the mechanical testing and modeling of brain tissue. Arch. Comput. Methods Eng. **27**, 1187-1230. (10.1007/s11831-019-09352-w)

[16] Zhao W, Choate B, Ji S. 2018 Material properties of the brain in injury-relevant conditions–experiments and computational modeling. J. Mech. Behav. Biomed. Mater. **80**, 222-234. (10.1016/j.jmbbm.2018.02.005) 29453025 PMC5841256

[17] Gent AN, Suh JB, Kelly III SG. 2007 Mechanics of rubber shear springs. Int. J. Non-Linear Mech. **42**, 241-249. (10.1016/j.ijnonlinmec.2006.11.006)

[18] Horgan CO, Murphy JG. 2010 Simple shearing of incompressible and slightly compressible isotropic nonlinearly elastic materials. J. Elast. **98**, 205-221. (10.1007/s10659-009-9225-1)

[19] Ogden RW. 1997 *Nonlinear elastic deformations*. Ellis Horwood, Chichester (1984). Reprinted by Dover, New York.

[20] Varga OH. 1966 Stress-strain behavior of elastic materials. New York, NY: Interscience.

[21] Gent AN, Lindley PB. 1958 Internal rupture of bonded rubber cylinders in tension. Proc. R. Soc. Lond. A **249**, 195-205. (10.1098/rspa.1959.0016)

[22] Janmey PA, McCormick ME, Rammensee S, Leight JL. 2007 Negative normal stress in semiflexible biopolymer gels. Nat. Mater. **6**, 48-51. (10.1038/nmat1810) 17187066

[23] Destrade M, Gilchrist MD, Murphy JG, Rashid B, Saccomandi G. 2015 Extreme softness of brain matter in simple shear. Int. J. Non-Linear Mech. **75**, 54-58. (10.1016/j.ijnonlinmec.2015.02.014)

[24] Horgan CO, Murphy JG. 2012 A boundary-layer approach to stress analysis in the simple shearing of rubber blocks. Rubber Chem. Tech. **85**, 108-119. (10.5254/1.3672433)

